# Sun Protection Policy in Elementary Schools in Hawaii

**Published:** 2004-06-15

**Authors:** Jay Maddock, Paul Eakin, Angela Techur-Pedro, Raphael Kaliko, D. Christian Derauf

**Affiliations:** Department of Public Health Sciences, University of Hawaii; Department of Pediatrics, John A. Burns School of Medicine University of Hawaii at Manoa; Department of Public Health Sciences, University of Hawaii at Manoa; Department of Public Health Sciences, University of Hawaii at Manoa; Department of Pediatrics, John A. Burns School of Medicine, University of Hawaii at Manoa

## Abstract

**Introduction:**

Childhood sun exposure is a major risk factor for skin cancer, the most common form of cancer in the United States. Schools in locations that receive high amounts of ultraviolet radiation have been identified as important sites for reducing excessive sun exposure.

**Methods:**

The objective of this study was to determine the prevalence of sun protection policies, environmental features, and attitudes in public elementary schools in Hawaii. Surveys were sent to all (n = 177) public elementary school principals in Hawaii. Non-respondents were called three weeks after the initial mailing. The survey asked about sun protection policies, environmental features, and attitudes toward sun protection. The survey was designed to measure all seven components of *Guidelines for School Programs to Prevent Skin Cancer*, issued by the Centers for Disease Control and Prevention.

**Results:**

Seventy-eight percent of schools responded to the survey. Only one school had a written school policy. Almost all schools (99.3%) scheduled outdoor activities during peak sun hours. School uniforms rarely included long pants (6.5%), long-sleeved shirts (5.1%), or hats (1.5%). Current policies did not support or restrict sun protection habits. Almost one third of those surveyed were in favor of a statewide policy (28.1%), and most believed excessive sun exposure was an important childhood risk (78.9%), even among non-white students (74.5%).

**Conclusion:**

Results of this study suggest the following: 1) school personnel in Hawaii are concerned about childhood sun exposure; 2) current school policies fail to address the issue; 3) most schools are receptive to developing sun protection policies and programs; and 4) students appear to be at high risk for sun exposure during school hours.

## Introduction

In the United States, the incidence of skin cancer is increasing faster than that of any other type of cancer (1). More than 1 million cases of skin cancer occur every year in the United States, nearly the same number as all other cancers combined (2). Basal cell carcinoma and squamous cell carcinoma are the most prevalent types of skin cancer but are the most curable. These two cancers accounted for almost 1.3 million new cases in the United States in 2002 (3). Melanoma, while much less prevalent, has a higher mortality rate, accounting for 75% of all skin cancer deaths (3). In Hawaii, melanoma incidence rates are similar to the nation at 20.5 per 100,000 for males (compared to 19.0 per 100,000 for the United States) and at 10.3 per 100,000 for females (compared to 12.0 per 100,000 for the United States) (4). Melanoma incidence rates among white males in Hawaii are rising rapidly from less than 30 per 100,000 between 1975 and 1979 to 62.8 per 100,000 from 1995 to 2000 (4).

Estimates show that the majority of lifetime sun exposure takes place during youth and that 50% to 80% of lifetime cumulative sun exposure occurs prior to age 18 (5). In addition, blistering sunburns prior to age 20 have been associated with an increased risk of developing malignant melanoma (6). Exposure early in childhood appears to be particularly important, with one study showing that children aged nine to 10 sustain more sun exposure than adolescents aged 14 to 15 (5). Another study using ultraviolet (UV) light-sensitive badges in six schools in England showed that primary school students had higher levels of exposure than secondary school students (7). Furthermore, consistent use of sunscreen with a sun protection factor of 15 throughout childhood and adolescence may reduce the lifetime incidence of basal and squamous cell carcinomas by 78% (5).

In the United States, Hawaii is the only state located in the tropics. Additionally, most Hawaii residents have a lifestyle that emphasizes outdoor activities. Hawaii's tropical location results in more direct UV radiation from the sun than non-tropical locations; research has shown that as latitude decreases, measured UVB radiation and melanoma incidence increases (8). Hawaii has a multiethnic population, but skin cancer occurs in all ethnic groups, especially in tropical climates (9). Also, due to Hawaii’s warm climate, schools often are built with sprawling, open layouts to take advantage of cooling trade winds. Because of this, students are frequently exposed to direct sunlight when walking between classes or during recess and physical education class. Several studies have been conducted evaluating institutional sun protection policy, including observational studies of children at daycare (10) and a national survey of elementary schools (11). In Hawaii, skin cancer prevention practices have been studied (12), and public health interventions in outdoor recreational environments have been able to show modest improvements in children’s sun protection behaviors (13-15), but no studies have been published on sun exposure or protection in schools. In the United States, only 12 states require education about skin cancer prevention at the elementary school level; Hawaii is not one of these states (16).

Sun exposure among children in the United States is common. In a national study of children aged six months to 11 years, children spent a median of 20 hours per week outdoors, including 10 hours while at school (17). Sunscreen (61.8%) and shade (26.5%) were the most common methods of sun protection in this study (17). Parents of children aged five to 10 at pools in Boston and Hawaii reported similar incidence of sunburn (40%) among their children during one summer (18). In this sample, sunscreen was the most widely used form of sun protection, with very few children wearing sunglasses or hats (18).

In April 2002, the Centers for Disease Control and Prevention (CDC) released *Guidelines for School Programs to Prevent Skin Cancer* (19). This document outlines seven recommendations for skin cancer prevention in schools:

1. Establish policies to reduce sun exposure.
2. Provide environmental supports for sun protection.
3. Provide health education on sun safety.
4. Involve the family in sun safety.
5. Provide professional development for staff for sun protection.
6. Support sun safety with health services.
7. Evaluate the effectiveness of these efforts.

With these seven guidelines in mind, we developed a survey to create a baseline measure of how well public schools in Hawaii were meeting these recommendations. We hypothesized that 1) elementary school children in Hawaii receive a significant amount of sun exposure during school hours, and 2) school policies rarely attempt to limit sun exposure or teach about the dangers inherent to sun exposure.

## Methods

In September 2002, a list of all public elementary schools (n = 177) in the state of Hawaii was obtained from the State Department of Education. Elementary schools were defined as schools that contained the first through fifth grades. Eight schools (4.5%) also included the seventh and eighth grades and were included in the sample. Students in sixth grade and kindergarten were included in approximately half of the schools, depending on the school complex. Private schools were not included in the study because of the difficulty in obtaining a sample that adequately represented charter schools, home schools, and other small schools (with less than 50 students), which are prevalent in Hawaii.

The 26-item survey queried current sun protection policies, amount of time students spent outside during peak sun hours, the use of sunscreen and sun-protective clothing by students and staff, and attitudes about the importance of sun protection. It was designed to measure all seven components of the CDC guidelines: policy; environmental change; education; family involvement; professional development; health services; and evaluation (19). Survey items were based on previous studies in the United States (11) and Australia (20). Additional items were generated by the study team. The survey instrument is available in the Appendix. All procedures were approved by the University of Hawaii's Institutional Review Board and the Hawaii Department of Education.

The survey was pre-tested with a convenience sample of seven elementary school administrators to determine readability and face validity of the instrument. Seven school administrators who had agreed to participate in a larger study to develop observational methods for sun protection among elementary school children were mailed a survey and then visited by a trained research staff member one to two weeks later. The staff member completed a structured interview with each school administrator following the written survey and assessed the comprehension of each question. None of the administrators reported any problems in understanding or completing any of the questions. Since no data were changed on these forms, the survey responses were pooled with all other responses.

During October 2002, cover letters explaining the study, survey, and return postcards were mailed to all remaining public elementary schools (n = 170) in the state of Hawaii. The surveys were returned anonymously; a return postcard sent by participants under separate cover identified schools that had completed the survey. Non-respondents were contacted by telephone three weeks after the initial mailing and encouraged to participate.

Data analysis was conducted using SPSS 11.5. Data were analyzed primarily by calculating percentages. For questions using a 5-point Likert scale, respondents who endorsed an item with a 4 or 5 were coded as agreeing with the statement. Schools were also grouped into either having a high enrollment (more than 40%) or a low enrollment (less than of 40%) of white students. Mean differences in the endorsement of items were assessed using *t*-tests.

## Results

Overall, 78% (n = 138) of the schools responded to the survey. Most of the respondents were principals (59%), vice principals (9%), or administrators (14.9%). Schools reported substantial ethnic diversity of their student bodies, with an average of 21.2% white, 26.9% Asian, 26.7% Native Hawaiian, and 10.7% Pacific Islander. Only 15.9% of schools that reported the ethnic composition of the student body reported 40% or more white students. The postcard was returned by 110 (62.1%) of the schools. Return of postcards was similar for Oahu (61.8%) and the neighbor islands (64.8%), (χ^2^[1] = .147, *P* = .70).

Table 1 presents results of the school survey. Only one school (0.7%) reported having a written policy to limit student sun exposure. However, 28.1% of schools believe that a statewide policy is needed. Of the 14.5% of schools with uniforms, only 1.5% include hats, 6.5% long pants, 5.1% long skirts, and 5.1% long sleeves as protective clothing options. Many schools allow students to wear protective clothing when outside. Hats (86.9%), sunglasses (72.9%), and sunscreen (98.5%) are allowed by most schools, and only 6.7% of schools require a doctor’s note to bring sunscreen to school. Very few schools provide sunscreen on field trips (4.3%).

Almost all (99.3%) of schools schedule outdoor activities between 10 AM and 2 PM, with 28.3% of schools scheduling at least half of their outdoor activities during this time. Shade-producing structures are common among schools (75.4%), but most (81.8%) cover less than 25% of play areas. Less than one quarter (22.2%) of school personnel reported that they often or always practice sun protection behaviors when supervising outdoor activities.

Less than half (47.7%) of schools teach sun protection as part of the health education curriculum. Almost half (48.9%) of schools have some students expressing concern about excessive sun exposure. Most schools (85.2%) are interested in interactive training sessions on the dangers of sun exposure. Only one fifth (20.1%) of schools send information home to parents about keeping their children sun-safe.

Less than 30% of schools reported that they provide any training in sun protection practices to physical education (PE) teachers, school administrators, and teachers. More than half of schools (53.6) reported having school nurses trained or knowledgeable about sun protection behaviors, but only 12.1% of schools reported that these nurses always or often instruct students about practicing sun protection behaviors. Only 6.6% of respondents had seen the CDC school guidelines, while 87.4% were interested in receiving a copy.

Most respondents (78.9%) believed that excessive sun exposure during childhood is an important health concern, and 74.4% believed this is also true for non-white students. However, only 19.8% of schools believed that they have better than average measures to protect their children from the sun.

No significant differences were seen between schools with a high proportion of white students (more than 40%) and schools with a low proportion (less than 40%) with regard to 1) the need for a statewide policy, instruction on sun protection behaviors, or information sent home to parents; 2) the perception that the school has adequate measures in place; and 3) the number of students expressing concern about sun exposure. A non-significant trend (*P* = .06) exists with schools with a higher percentage of white students believing that excessive sun exposure is an important health concern (Table 2).

## Discussion

Among public elementary schools in Hawaii, we found an absence of policies to reduce sun exposure and a lack of knowledge about the CDC guidelines to prevent skin cancer. We found that few teachers receive professional development in sun protection practices, and that uniform policies do not usually require protective clothing. Most current school policies do not prohibit or encourage sun protection behaviors, and most administrators stated that they had never thought about the effects of sun exposure on students during school time. Despite these results, administrators are largely in favor of stronger policies and believe sun exposure is an important health issue. Our results suggest the need for state education departments to develop sun protection policies, environmental supports, and a sun protection curriculum.

Limitations of our study include the selection bias inherent to survey studies and the possibility that the person responding to the survey is not well-informed about school conditions. A further study is currently underway that will address some of these issues through site visits and direct observation of students’ sun protection behaviors.

Our results are similar to the study done in 1997 by Buller et al in which only 3.4% of schools in the United States reported written sun protection policies, while 76.4% of principals were willing to make environmental changes (11). The Buller study, however, had several methodological limitations, including a low response rate (41%) and a high proportion of schools in cities with low UV intensity (63%). Our findings are likely to be similar to findings in other states that do not have a comprehensive statewide or district-wide policy, because administrators appear to be largely in favor of sun protection measures but have not made them a priority for their schools.

The results from Hawaii and the United States contrast starkly with results from Australia. In 1993, the Victoria Anti-Cancer Council developed a SunSmart school accreditation program to recognize schools for having comprehensive sun protection policies (20). From 1992 to 1997, sun protection policies in Victoria increased from 17% of schools to 76% (20,21). Sun protection practices are also much more rigorous in Victoria, with 78% of schools recommending broad-brimmed hats, 96% providing sunscreen or encouraging parents to supply it, and 93% teaching sun protection in classes (20). The CDC has recommended that school districts conduct periodic evaluations to assess how well schools are meeting the guidelines (19). To our knowledge, this study represents the first attempt to evaluate the sun protection policy and environment of all public elementary schools in a state. Given the substantial lifetime sun exposure burden encountered during the elementary school years, sun protection policies have the potential to significantly alter an individual's risk for later development of skin cancers as adults. Results from this study and from programs in Australia are encouraging because most school principals have a positive outlook on sun protection policies. The development of an accreditation program by a national or state group could lead to great changes in elementary school sun protection practices. The *Guidelines for School Programs to Prevent Skin Cancer* should also be widely distributed along with model policies to schools — especially to district- and state-level administrators — to encourage their adoption.

## Figures and Tables

**Table 1:** Existing School Policies and Education on Sun Protection, Public Elementary Schools, Hawaii, 2002

**Policies**	**% Respondents (n = 138)**
Schools with written policy that limits students' exposure to sunlight during outdoor activities	0.7

Schools that feel statewide policy to limit sun exposure to children during school is needed	

5 - Very much	11.1
4-	17.0
3 - Average	28.9
2-	25.2
1 - Not at all	17.8
Schools that allow the use of sunscreen during outdoor activities	98.5
Schools that require doctor's prescription or note for student to use sunscreen lotion	6.7
Schools that allow the use of hats during outdoor activities	86.9
Schools that allow students to wear sunglasses when outdoors	72.9
Schools that enforce a uniform policy	14.5

Uniform options among schools with uniform policies	

Hats	1.5
Long pants	6.5
Long skirt	5.1
Long sleeves	5.1

**Environmental Support**	

Schools have outdoor activities between 10 AM and 2 PM	99.3

Percent of activities that fall between 10 AM and 2 PM	

< 25%	51.5
26-50%	20.1
51-75%	13.4
> 75%	14.9

Percent of play or activity covered by shade	

< 25%	81.8
26-50%	12.4
51-75%	3.6
> 75%	2.2
Schools that provide sunscreen for students during school field trips	4.3
Schools that provide shade-producing structures	75.4
Schools who claim that students are concerned about excessive sun exposure during outdoor activities	48.9

School personnel who practice sun protection behaviors while supervising outdoor activities	

Always	3.7
Often	18.5
Sometimes	52.6
Seldom	21.5
Never	3.7

**Education**	

Percent of schools that teach sun protection as part of the health education curriculum	

Yes	47.7
No	47.7
Not sure	4.7

**Family Involvement**	

Schools that send home information to parents about sun safety	20.1

**Professional Development**	

Staff/teachers trained or knowledgeable about sun damage and sun protection practices	

Physical education teachers	27.5
Administrators	21.7
School nurses	53.6
Teachers	24.6
Others	8.0

School staff had seen CDC sun protection guidelines	

Yes	6.6
No	92.7
Don't Know	0.7

**Health Services**	

School health nurses routinely instruct students on sun protection behaviors	

Always	3.0
Often	9.1
Sometimes	50.0
Seldom	25.0
Never	12.9

Attitudes toward sun exposure	

Schools that believed that excessive sun exposure during childhood is an important health concern	78.9
Schools that believed that excessive sun exposure during childhood is an important health concern for **non-white children**	74.4
Schools that believed they have better than average measures to protect their children from the sun	19.8

**Table 2:** Results of Survey on Sun Protection Policies Among Public Elementary Schools With a High and Low Proportion of White Students, Hawaii, 2002

	**Low ** **(< 40%) White Population** **(n = 111)** a	**High ** **(> 40%) White Population** **(n = 21)**	

**Survey Question**	**Mean (SD)**	**Mean (SD)**	***t* value**
A statewide policy to limit sun exposure in schools is needed	2.84 (1.24)	2.85 (1.31)	*t*_127_ = -0.02, *P* = .98
School personnel instruct students on sun protection behaviors	2.68 (0.90)	2.81 (0.98)	*t*_124_ = -0.61, *P* = -.54
School sends information home to parents on sun protection behaviors	1.79 (0.41)	17.6 (0.44)	*t*_126_ = 0.33, *P* = .75
Schools teach sun protection as part of the curriculum	1.58 (0.59)	1.55 (0.60)	*t*_122_ = 0.19, *P* = .86
Amount of concern that students express about sun exposure	1.65 (0.71)	1.65 (0.81)	*t*_126_ = -0.01, *P* = .99
Perception that school has adequate measures to limit students' sun exposure	2.86 (1.00)	2.62 (1.12)	*t*_129_ = 0.99, *P* = .32
Perception that excessive sun exposure is an important health concern	4.05 (0.91)	4.36 (0.73)	*t*_129_ = -0.15, *P* = .14
Perception that excessive sun exposure is an important health concern among non-white students	4.13 (0.85)	4.50 (0.74)	*t*_130_ = -1.88, *P* = .06
School personnel practice sun protection behaviors while outside	3.03 (0.82)	3.10 (0.64)	*t*_127_ = -0.37, *P* = .71

a Six schools did not report student body ethnicity.

b All survey questions used a 5-point Likert scale, with 4 or 5 coded as agreeing with the survey statement.

## Appendix

**Table:**

For each question please mark the one answer that most closely represents the situation for your school.					
1.	Is your school public or private?				
	Public Private				
2.	What is your position at your school? ______________________________				
3.	Does you school have a written policy that attempts to limit your students' sun light exposure?				
	Yes No				
4.	Does your school have outdoor activities scheduled between the hours of 10 AM and 2 PM? (e.g., PE classes, recess)				
	Yes No				
	If yes, what percent of outdoor activities fall during this time?				
	A. Less than 25% B. Between 26-50% C. Between 51-75% D. More than 75%				
	If no, please go to number 3.				
5.	Do your outdoor activity areas contain shade-producing structures such as trees or roof overhangs?				
	Yes No				
6.	What percentage of your play/activity area is covered by shade?				
	A. Less than 25% B. Between 26-50% C. Between 51-75% D. More than 75%				
7.	Does your school utilize mandatory uniforms?				
	Yes (please answer number 8) No (skip to question 9)				
8.	Does the school uniform include any of the following options? (Please circle all that apply).				
	Long sleeves Hats Long pants Long skirts				
9.	Do the school personnel who supervise outdoor activities use sun protection behaviors such as wearing a hat, long sleeves or umbrellas the majority of the time?				
	Always		Sometimes		Never
	5	4	3	2	1
10.	Does your school allow students to wear hats when outdoors?				
	Yes No				
	If not, why not?				
11.	Are students allowed to wear sunglasses during outdoor activities at school?				
	Yes No				
	If not, why not?				
12.	Does your school allow students to wear sunscreen when outdoors?				
	Yes No				
13.	Does your school provide sunscreen for students to use during school field trips?				
	Yes No				
14.	Would the use of sunscreen by a student require a note from their doctor?				
	Yes No				
15.	To what extent do you feel that excessive sun exposure during childhood is an important health concern?				
	Very much		Average		Not at all
	5	4	3	2	1
16.	To what extent do you feel that excessive sun exposure during childhood is an important health concern in non-white students?				
	Very much		Average		Not at all
	5	4	3	2	1
17.	Do you feel that your school has adequate measures in place to limit the amount of sun exposure students receive during the school day?				
	Very much		Average		Not at all
	5	4	3	2	1
	Please explain why or why not you have these measures in place:				
18.	Are you aware of students expressing concern about excessive sun exposure during school activities to you or to other faculty?				
	Very much		Average		Not at all
	5	4	3	2	1
19.	Does your school teach sun protection as part of the health education curriculum?				
	Yes No				
	If yes, in which grades?				
20.	Does your school send home information to parents about keeping their children sun safe?				
	Yes No				
21.	Are any of the following staff members routinely trained in sun protection practices? (Circle all that apply)				
	Teachers School administrators PE teachers School nurses Others				
22.	Do the school health nurses routinely instruct students on sun protection behaviors?				
	Always		Sometimes		Never
	5	4	3	2	1
23.	Would you be interested in having us give an interactive session with your students about the dangers of sun exposure?				
	Very much		Average		Not at all
	5	4	3	2	1
24.	Do you feel that a statewide policy attempting to limit sun exposure to children during school is needed?				
	Very much		Average		Not at all
	5	4	3	2	1
25.	The Centers for Disease Control and Prevention recently published guidelines for schools to help prevent skin cancer. Have you seen this document?				
	Yes No				
26.	Would you like to receive a copy of this document?				
	Yes No				
This is the end of the survey. Thank you very much for your time and effort in completing this survey.					
